# Caliper measurement to improve clinical assessment of palpable neck lumps

**DOI:** 10.1308/003588412X13373405386178

**Published:** 2012-09

**Authors:** Peter Brennan

**Affiliations:** Queen Alexandria Hospital, Portsmouth,UK

## Abstract

**Wasson J, Amonoo-Kuofi K, Scrivens J, Pfleiderer A** Caliper measurement to improve clinical assessment of palpable neck lumps. *Ann R CollSurgEngl* 2012; **94**: 256–60

I read the above paperwith interest. It is certainly an easy to use technique to monitor the size of readily palpable lumps that are seen by head and neck specialists and would seem to increase the accuracy of clinical measurement.However, I was concerned that the authors stated that as a result of increasing numbers of referrals not all new patients with a palpable neck lump will go on to have an ultrasound and that calipers can improve clinical assessment, particularly when an ultrasound machine is not available.

They also mentioned that all patients with a lump greater than 9mm in their unit will go on to have an ultrasound. The authors make no mention of what the upper limits of normal size for lymph nodes are in various levels of the neck; these vary depending on site. For example, a 15mmjugulodigastric node with a short axis on ultrasound less than 9mm may well be reactive, while a similar size node in the submental area is almost always pathological and requires fine needle aspiration cytology to exclude malignancy.^1^

The additional advantage of ultrasound is that it can confirm a reactive node at the first visit not only by short-axis measurement but also by demonstrating normal hilar architecture and blood flow using colour flow Doppler. None of these assessments can be made using clinical examination or calipers and therefore patients having clinical assessment alone will undoubtedly be followed up in a review clinic instead of being reassured and discharged.

Therefore, perversely, not having access to ultrasound may result in additional clinic visits as well as potentially delaying a malignant diagnosis irrespective of better accuracy in determining the lymph node size using calipers. In addition to diagnosing metastatic disease, lymphoma nodes (which in certain subtypes can remain small for some time) often have readily visualised ultrasound appearances and rapid diagnosis can be made using ultrasound guided tru-cut biopsy.^2^

Finally, the authors make no mention of oral and maxillofacial surgeons (OMFS) managing neck lumps. In many units in the UK, both ENT and OMFS work together to provide a high-quality neck lump service with a head and neck radiologist; many patients can be discharged at the first visit following clinical assessment and ultrasound.
